# Freeze-Dried Royal Jelly Proteins Enhanced the Testicular Development and Spermatogenesis in Pubescent Male Mice

**DOI:** 10.3390/ani9110977

**Published:** 2019-11-15

**Authors:** Zhicheng Shi, Hamdard Enayatullah, Zengpeng Lv, Hongjian Dai, Quanwei Wei, Lirong Shen, Babrak Karwand, Fangxiong Shi

**Affiliations:** 1 College of Animal Science and Technology, Nanjing Agricultural University, Nanjing 210095, China; 2017105023@njau.edu.cn (Z.S.); 2017105117@njau.edu.cn (H.E.); lvzengpeng@njau.edu.cn (Z.L.); 2018105021@njau.edu.cn (H.D.); weiquanwei@njau.edu.cn (Q.W.); 2 College of Food Science, Zhejiang University, Hangzhou 310058, China; shenlirong@zju.edu.cn; 3 Faculty of Veterinary Science, Kunduz University, Kunduz City 0093, Afghanistan; babrakkarwand100@gmail.com

**Keywords:** royal jelly, development, pubescent, disruption, testis

## Abstract

**Simple Summary:**

Spermatogenesis and hormones secretions are serious life-threating and complicated process, which can be improve through science-based approaches. Royal jelly is a thick white milky fluid secreted by the hypopharyngeal and mandibular glands of young nurse worker bees (Apis mellifera) and used to feed their queen to expand their life. The results of the study revealed that, the growth performance of testis in exposed mice to freeze-dried Royal Jelly for 35 consecutive days were significantly enhanced in moderate dose among other treated doses. However, at Post Natal Days (PNDs 14 and PNDs 21), obviously changes were observed in histological examination of the testis while at PNDs-07 no major changes were observed. The Tunnel assay showed that, less apoptotic cells were detected in the testis of mice in high dose of freeze-dried RJ and oral administration of freeze-dried royal jelly can aggravate adverse effects via tempestuous on sexual hormone secretion at both PNDs 21 and PNDs 35 respectively.

**Abstract:**

Spermatogenesis and hormones secretions are crucial endocrine and physiological process for maintaining the life. Royal Jelly (RJ) bioactive components are Major Royal Jelly Proteins (MRJPs), owing exceptional biological properties. However, the effects of RJ on pup’s testicular development during neonatal and pubertal period exposure hasn’t been adequately studied. The aim of the study was to detect neonatal sexual hormones concentration and histopathological changes on testicular development of the male progeny after oral exposure to freeze-dried RJ for 35 consecutive days. After mice give birth, male pups were collected together, separated by sex, and randomly standardized to seven (7) male pups per dam. Male pups were assigned to control diet (CON group), low dose RJ (L-RJ group) diet (control diet + 125 mg/kg/day RJ), moderate dose RJ (M-RJ group) diet (control diet + 250 mg/kg/day RJ) and high dose of RJ (H-RJ group) diet (control diet + 500 mg/kg/day RJ). After weaning, male pups were continuously fed with freeze-dried RJ until the age of PNDs 35. The results revealed that, oral M-RJ (250 mg/kg/day) administration significantly (*p* < 0.05) increased the testis weight, the diameter of seminiferous tubule and the height of seminiferous epithelium of offspring mice at PNDs 14. However, high-dose RJ (500 mg/kg/day) decreased the diameter of seminiferous tubule but increased the height of seminiferous epithelium of male offspring (*p* < 0.05) at the same time point. Furthermore, oral M-RJ treatment significantly (*p* < 0.05) increased the testis weight and spermatogenesis at PNDs 21. Whereas, oral H-RJ treatment significantly (*p* < 0.05) reduced the diameter of seminiferous tubule and the height of seminiferous epithelium at PNDs 21. At PNDs 35, oral M-RJ treatment increased the testis weight, the diameter of seminiferous tubule and the level of FSH. While, high-dose of RJ reduced testis weight and size (diameter of seminiferous tubule and height of seminiferous epithelium), ratio of apoptotic germ cells and incomplete spermatogenesis collectively. In addition, sexual hormone secretions (FSH, LH, E2) were decreased after RJs treatment (L-RJ, M-RJ, H-RJ) at PNDs 21 respectively. In conclusion, the results concluded that oral administration of low and moderate doses of RJ could enhance the development of testis at neonate period until pubescent, whereas unfavorable adverse effects induced by high dose of RJ might remain.

## 1. Introduction

Bee products have been used since many decades ago to treat various human diseases. Royal Jelly (RJ) well-known bioactive components are Major Royal Jelly Proteins (MRJPs), owing exceptional biological properties and considered as potential factor for expanding honeybee life. Analysis of RJ proteins showed that 82–90% (*w*/*w*) is constituted by MJRPs and RJ is contained of some essential free amino acids and complex proteins of MRJP family, essential for nourishing both the queen bee and larvae [1,2]. RJ is also called “Bee Milk” and the fresh royal jelly is slightly ropy secreted from the hypopharyngeal and mandibular salivary glands of young nurse (Apis mellifera) aged between 5–14 days [3,4]. RJ is consumed worldwide in diet for its beneficial nutritive, protective, antioxidant and anti-inflammatory effects [5]. At the meantime, China is the largest producer and consumers of RJ in the world [6]. Some studies suggest that RJ has estrogenic activity which was similar to other exogenous steroid hormones including testosterone and 17β-estradiol [7,8]. These estrogen-like compounds have been extensively reported to be similar to estrogens and they could exert various estrogenic or anti-estrogenic effects in the reproductive systems through modulating the estrogen receptors (ERs) [9]. Exogenous estrogen or estrogen-like compounds can be found in many plant and animal source materials such as seeds, vegetables, milk, and dairy products [9,10]. Meanwhile, RJ was reported to inhibit the negative effects affected by exogenous estrogen on male reproductive system [7]. According to reproductive relevant studies, RJ could expose estrogenic effects in vivo and in vitro [11,12]. Whereas, the effect of these estrogen-like compounds on male reproductive system remain unclear, particularly on neonatal period. Hence, it is very significant to investigate the reproductive function of offspring when constantly exposure to RJ from neonatal to maturity period.

Considering the previous studies, RJ has many pharmacological activities such as anti-inflammatory [13] antioxidant [14], antimicrobial [15], anti-tumor [16], and immune-modulatory function [17]. Moreover, previous reports have shown that RJ can be effective in enhancing male hormones and sperm count as well as reproductive toxicities reduction [18,19]. It has been reported that, RJ treatment (by gavage 100 mg/kg/day) improved epididymal sperm motility and in vitro fertilizing capacity in adult male mice [20]. However, some study revealed that RJ could improve oxidative stress, male infertility [18] and might inhibit the cell proliferation by breaking E2-induced signaling [7]. Additionally, early research illustrated that RJ (800 mg/kg/day) treatment could increase sperm deformity rate and serum hormones (testosterone) level of the male rats [21]. Hence, RJ may have a safety period to predict consumers’ health risks which is supposed to have a tremendous fascination for human health and hasn’t addressed so far. However, RJ is enriched biological product containing very complicated active elements which cover majority of the nutritional elements required by the growth of the human body [11,13,22,23]. The unique feature of RJ is having the active substance of 10-hydroxy-trans-2-decenoic acid (HDEA), which is believed to be present only in RJ [24,25]. 

However, RJ need to be resorbed quickly to fulfill biological activity. Few studies have administrated neonatal mice with oral RJ because of its limited metabolic capacity. In a pilot study it has demonstrated that oral RJ mediated estrogen signaling and the disruption of hormonal balance may lead to abnormalities [26,27]. In addition, inclusive understanding of the components of RJ may improve the pharmaceutical and clinical uses of RJ as an alternative medication. Finally, in the recent decades, there have been an increasing number of studies investigating the effects of RJ on reproductive system of mice. To our knowledge, no study has evaluated the estrogenic like effects and adverse effects of RJ on testicular and spermatogenesis development in early age mice. Therefore, the objectives of this study were to detect neonatal sexual hormones concentration, histological changes of pups’ testis and spermatogenesis in different time points after oral exposure of freeze-dried RJ for 35 consecutive days.

## 2. Materials and Methods

### 2.1. Ethics Statement

All experimental procedures and protocol were designed and approved in accordance with the guidelines for the animal welfare and use of animals, prepared by the Institutional Animal Care and Ethical Committee of Nanjing Agricultural University (Permit number: SYXK (Su) 2019–0036), Nanjing, China. All animals were treated compassionately and with regard for alleviation of suffering, they produce complied guidelines for animal care. 

### 2.2. Animals 

Two-month-old male and female ICR mice (totally 40 mice; 20 males and 20 females) were obtained from Qinglongshan Laboratory Animal Company (Nanjing, China). After a week adaption to feeding, 2 male and 2 female mice were housed in ventilated cage with hardwood chip bedding under controlled conditions of temperature (21 ± 1 °C), lighting (12/12-hr light/dark cycle). Mice were fed with a standard laboratory diet and fresh water ad libitum. Female mice were separated and labelled once they got pregnant. After the mice give birth, male pups were collected together, separated by sex, and randomly standardized to seven (7) male pups per dam. Female pups were untreated and used as breeders after reaching adulthood. Experiment began at the first day of the male pups born. Freeze-dried RJ was freshly prepared with normal saline every day. A pipette was inserted compassionately inside each pup’s mouth for daily RJ administration. Doses were designed 125, 250 and 500 mg/kg/day, which marked “L-RJ”, “M-RJ”, “H-RJ” as well as control group “CON”. During the experiment, pups consumed the dose easily via pipette and did not show stress, weight loss, or any other gross toxic effects. At PNDs 7, six to seven pups were randomly selected using cervical dislocation under CO_2_ anesthesia. The same operations were carried out as above at PNDs 14, PNDs 21 and PNDs 35 respectively.

### 2.3. Preparation and Dissolving of Freeze-Dried Royal Jelly

Pure freeze-dried RJ was purchased from Hangzhou Biyutian Health Food Corporation, Ltd. (Tonglu, Zhejiang, China). According to manufacturer’s reports, it is the excretive mixture of nutrition gland and maxilla gland of the head of little worker bee. The RJ was produced from the purest beehives which were free from chemicals or antibiotic sprays. For preparing a saline dissolved extract of freeze-dried RJ stoke with concentration of 125 mg/kg/day, 250 mg/kg/day and 500 mg/kg/day, they were dissolved in 10 mL normal saline and freshly used at daily bases through gently pipetting into the pup’s mouth. The remaining freeze-dried RJ was then placed at 4 °C in the refrigerator.

### 2.4. Sample Collection

At PNDs 7, six to seven pups were randomly selected using cervical dislocation under CO_2_ anesthesia. Reproductive organs (testis and epididymis) and other organs (spleen, liver and kidney) were collected for weight measurements. For determination of acidity in the stomach, pH was measured in different time points. The same operations were carried out as above at PNDs 14, PNDs 21 and PNDs 35 respectively. Blood was collected at PNDs 21 and PNDs 35. The serum was centrifuged at 4000 r/min for 10 min and stored at −20 °C until use for the detection of hormones.

### 2.5. Histological Study of Testis

**Figure 1:**
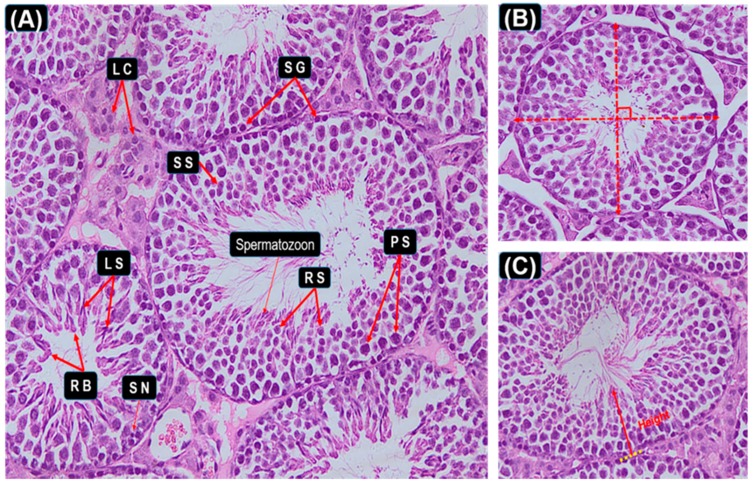
These combined handy reference figures show the hematoxylin and eosin stained images of control mice at the age of day 35. Above images were used as an example evaluated by light microscopic examination under 20× magnification. Different abbreviated markings and arrows indicate the following: Spermatids Cells (SC); ST-lumen, seminiferous tubule lumen; Residual Bodies (RB); Round Spermatids (RS); Secondary Spermatocytes (SS); Primary Spermatocytes (PS); Spermatogonia (SG); SC nuclei (Sertoli cell nuclei); Leydig Cells (LC). In addition, Figure 2C, the lines perpendicular to each other in red indicated our method of measuring diameter. Finally, different markings inside the images were inserted through Adobe Photoshop CS5.

To determine effects on reproductive system development, testis was collected and fixed in 10% cold neutral buffered formalin overnight and then changed to cold 70% ethanol. Tissues were then processed for histology, embedded in paraffin, and cut at 5-μm thickness. The obtained tissue sections were collected on glass slides, deparaffinized and stained by hematoxylin and eosin stain for histopathological examination. Five sections of testis from each of three different doses were observed for the histological changes in the seminiferous tubules as the previous method reported [28,29,30,31]. (Total of 30 sections were observed from each mouse). The diameter of a seminiferous tubule was defined as the shortest distance when the length of the outer edge of the tubule measured by two vertical measuring scales is equal (Figure 1B). The seminiferous epithelial height was defined as the shortest distance between the germ cell which is closest to the center of the lumen and the basement membrane (Figure 1C).

### 2.6. Reproduction Development Assesment 

The organization of the seminiferous tubule has been studied frequently in rodent species [32]. Each cell type of the cell association in cross-sectioned seminiferous tubules is morphologically integrated with the others in its developmental processes. Based on the examination of cross-sectioned seminiferous tubules, spermatogenesis and steps of spermatogenesis can be known clear [29,33,34,35]. Our assessment of spermatogenesis is based on Johnsen’s method [36]. Testicular morphology in all groups were observed by light microscope and represented the most frequently observed structural changes inside the seminiferous tubules. Briefly, the Scoring method applies a grade from 0 to 5 to each tubule cross section according to the following criteria (Table 1). The mean testis scoring was calculated for 100 seminiferous tubules per group.

### 2.7. Immune Histochemistry (IHC) Analysis 

The 5-μm paraffin tissue was serially dewaxed, sectioned, dehydrated and rehydrated in xylene and distilled water. After antigen retrieval by microwave irradiation into citrate buffer, endogenous peroxidase activity in sections was quenched by 3% hydrogen peroxide at room temperature for half hour and put inside PBS and then blocked in 3% BSA/PBS. The sections were then blocked with 5% bovine serum for 15 min. The sections were subsequently incubated with specific primary antibodies against PCNA (GB13030; Wuhan Saiweier Biological Technology, China) overnight at 4 °C in a humidified chamber (1:150 dilutions). The sections were then washed with PB, incubated with secondary antibody, and then stained by adding 3, 3-diaminobenzidine after being washed with PBS. Lastly, the sections were counterstained with hematoxylin and rinsed in tap water. IHC micrographs were observed and analyzed on an Olympus BX51 photomicroscope.

### 2.8. Tunnel Assay 

The level of apoptosis-related DNA fragmentation in the testicular was evaluated by TUNEL assay a commercially available kit BrightGreen Apoptosis Detection Kit (A112, Vazyme Biotech, Nanjing, China). The sections of 5-μm paraffin tissue specimens were serially sectioned, and the standard method was employed according to the manufacturer’s instructions. The DAPI agent was used to visualize the apoptotic germ cells, and sections were observed under a light microscope. The cells in the test exhibiting green nuclear staining were considered positive for nuclear DNA fragmentation. Data collected using a Nikon DS-U3 system and analyzed using Image-Pro Plus6.0 software. Ten (10) independent fields of vision were chosen as representative fields in each group mice.

### 2.9. Hormones Determination in PNDs 21 and PNDs 35 

The level of serums hormones (FSH, LH, T and E2) in each group at PNDs 21 and PNDs 35 were measured by commercial radio immunoassay (RIA) kits (Nanjing Jiancheng Bioengineering Institute, Jiangsu, China) according to the manufacturer’s instructions. 

### 2.10. Statistical Analysis 

Computations were carried out with SPSS (Version 20.0) and Graph Pad Prism (Version 5.0). All values were expressed as mean ± standard error of the mean (SEM). The differences across groups were calculated with one-way analysis of variance (ANOVA), *p* < 0.05 were considered significant.

## 3. Results

### 3.1. Grwoth Performance 

After the oral exposure to freeze-dried royal jelly for 35 consecutive days, the body weight of treated groups mice was increased gradually by week until they were sacrificed. The average body weight in this experiment increased from 1.5 g at day 1 to 28.5 g at day 35 in control and treated groups. However, the data showed no statistical significantly different among the treated and CON groups respectively (Figure 3).

### 3.2. Organs Weight Measurment 

The organs weight was measured at 4 different Post-Natal Days (PNDs 07, 14, 21, 35). Generally, the spleen weight of mice in L-RJ and M-RJ group were significantly (*p* < 0.05) decreased compared with CON groups at PNDs 07 (Table 2). Similarly, the weight of spleen was decreased significantly (*p* < 0.05) in H-RJ treatment compared with CON group at PNDs 14. However, oral moderate dose of royal jelly administration significantly (*p* < 0.05) increased the weight of testis, liver and kidney compared with the CON group (*p* < 0.05) at PNDs 14. Furthermore, orally supplying with M-RJ can increase the testis weight while the weight of liver and kidney were significantly decreased (*p* < 0.001) at PNDs 21 (Table 2). However, H-RJ treatment increased the weight of spleen and liver in PNDs 21. Meanwhile, the pH of stomach was decreased after L-RJ and M-RJ treatment (*p* < 0.05). In addition, at PNDs 35, the weight of spleen was increased compared with the CON group. Similarly, the pH of stomach was increased after RJ administration. Interestingly, in Table 2, the testis weight was increased after administration with moderate dose of royal jelly whereas the weight of testis decreased (*p* < 0.001) after high dose of royal jelly treatment at PNDs 35.

### 3.3. Seminiferous Epithelial Height and Seminiferous Tubule Diameter Measurements

**Figure 2:**
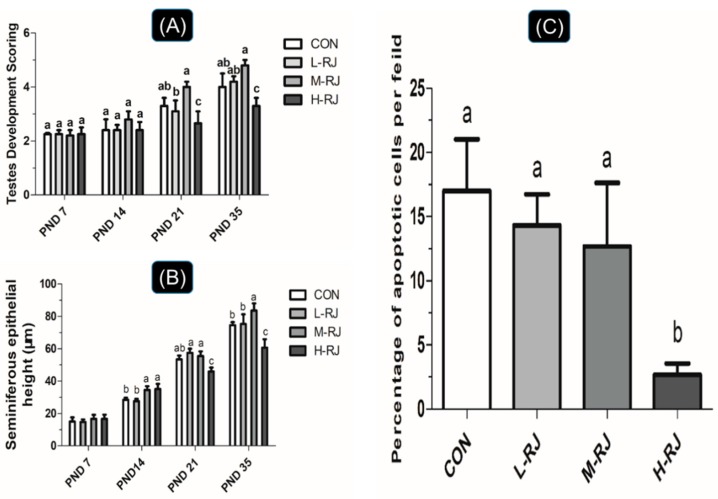
The data in graph represent testis scoring results and data shown as mean ± SE (n = 100) and different letters (**A**,**B**,**C**) within a column represent significantly (*p* < 0.05) differences among the groups.

To determine the effect of oral dietary RJ on the development of testis, the diameter of testis and height of seminiferous epithelial of male mice were evaluated at four different timing stages (PNDs 07, 14, 21 and 35). At PNDs 14, the diameter of the tubule and height of seminiferous epithelial of the mice were significantly increased (*p* < 0.001) after supplementation with moderate dose (M-RJ; 250 mg/kg/day) (Table 3). However, high dose of royal jelly (H-RJ; 500 mg/kg/day) decreased the height of seminiferous epithelial compared with the CON group at the same timing point (Figure 2B). Furthermore, administration with H-RJ significantly (*p* < 0.05) decreased the mean seminiferous tubules diameter and the height of seminiferous epithelial compared with the CON group at PNDs 21 (Table 3, Figure 2B). Additionally, Oral exposure to H-RJ for 35 consecutive days, the testis of the mice showed a reduction on its size (both epithelial height and diameter of seminiferous tubules) compared to CON group (Table 3, Figure 2B) respectively. Interestingly, the seminiferous epithelial height of mice was increased (*p* < 0.05) after M-RJ treatment (Figure 2B).

### 3.4. Histopathological Examination of the Testis

**Figure 3:**
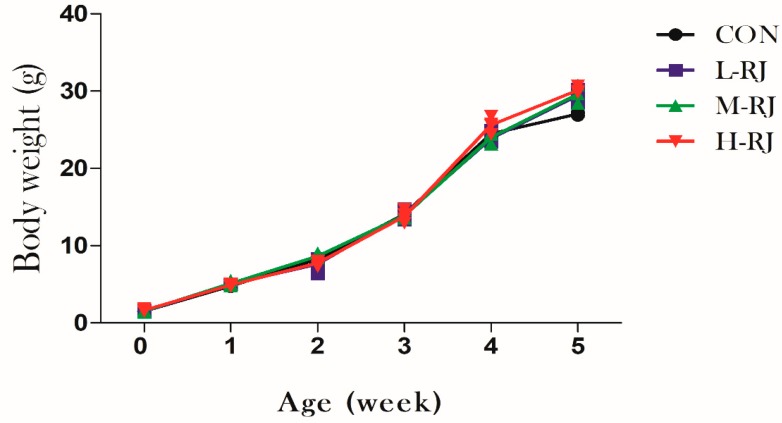
The effects of freeze-dried RJ on male mice body weight are shown in Figure 1. “CON” means the control group, “L-RJ”, “M-RJ” and “H-RJ” are the abbreviation of “low dosage of RJ”, “moderate dosage of RJ” and “high dosage of RJ”, respectively.

**Figure 4:**
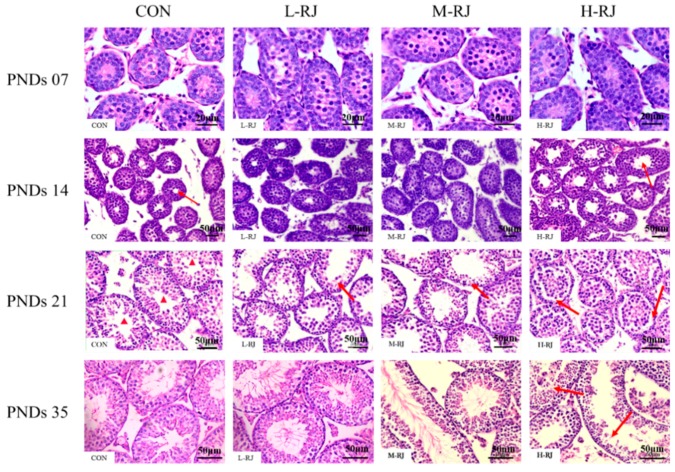
Effects of different doses of freeze-dried RJ on the histoarchitecture of seminiferous tubules at Post-Natal Days (PNDs 07, PNDs 14, PNDs 21 and PNDs 35) in mice. The images pasted with letter “CON” represent mice in control group, showing the normal features, the images pasted with letter “L-RJ”, ”M-RJ” and ”H-RJ” represent low dose of freeze-dried royal jelly treatment group, moderate dose of freeze-dried royal jelly treatment group and high dose of freeze-dried royal jelly treatment group, respectively. For each group and each stage, we captured three representative images. At PNDs 07, images were captured at the magnification of 40×. Bar 20 μm. Testis images of PNDs 14, PNDs 21 and PNDs 35 mice captured at the magnification of 20×. Bar 50 μm. Red arrows in PNDs 14 indicate the space of seminiferous tubules. Red arrows in PNDs 21 (L-RJ, M-RJ) indicate the seminiferous epithelium. Red arrows in PNDs 21 (H-RJ) indicate the abnormal space in the basal part of the seminiferous epithelium. Red arrows in PNDs 35 (H-RJ) indicate the un-synchronously seminiferous epithelium. Different markings inside the images were inserted through Adobe Photoshop CS5.

In the first histological examination at the stage of Post-Natal Days (PNDs 7). No obvious differences of the testis structures were observed between treatment and control groups Figure 3 (PNDs 07). At the stage of PNDs 14, as the red arrow showed, more lumen space of each seminiferous tubule was observed after high dose (H-RJ) (500 mg/kg/day) of freeze-dried royal jelly compared to control. At the stage of PNDs 21, significant histological changes were observed among the groups. The cross-sectioned of seminiferous tubules in CON group (Figure 4, PNDs 21) were composed of Sertoli cells, spermatogonia and spermatocytes. Seemingly few round spermatids were observed in H-RJ group Figure 4 (PNDs 21). However, there was none lumen space in seminiferous tubules of H-RJ group compare to CON group. Meanwhile, each cross-section of the seminiferous tubules was associated with different types of germ cells in a row. Particularly, spermatogonia maintained in its position and stick to the wall of the seminiferous epithelium after high dose of royal jelly treatment. Most spermatocytes (pachytene spermatocytes and leptotene spermatocytes) lost their sites (location) and moved to the center un-synchronously. As the Figure 4 showed, many round spermatids developed out of order and they were released to the center without deformation. At the PNDs 35, mice had a full complement of germ cell generations and the cycle of the spermatogenesis had formed. In addition, complete series of the cell associations were observed in a logical order especially in CON, L-RJ and M-RJ group respectively. Finally, as the single cross-sectioned seminiferous tubules showed in H-RJ group, the development of spermatogenesis of the testis was un-synchronously. Whereas, many spermatozoa released before other spermatids deformed. Figure 4 (PNDs 35, H-RJ group). 

### 3.5. Histopathological Stages and Characteristics of Germ Cell

**Figure 5:**
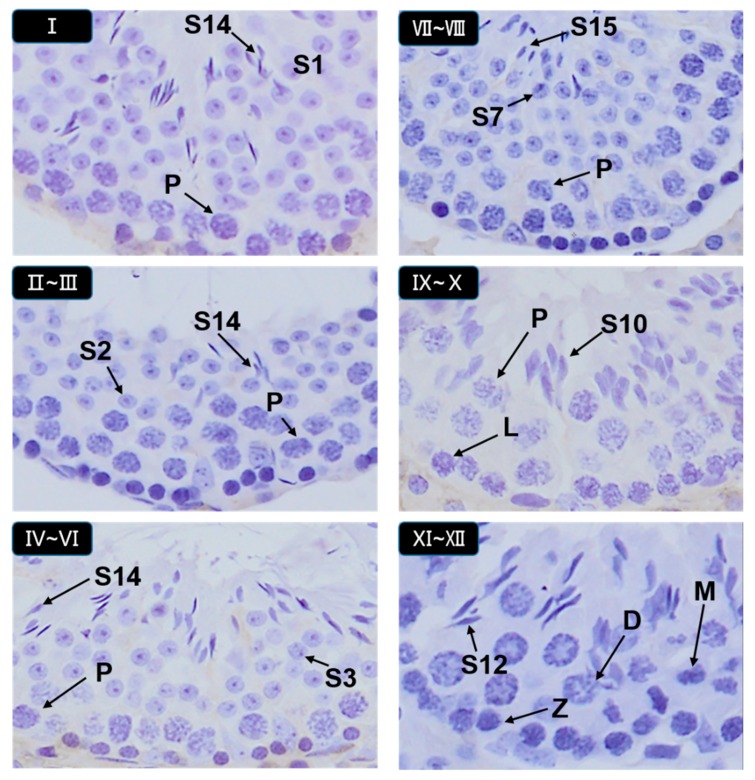
Hematoxylin staining of seminiferous epithelia at stages I–XII. These images were used as an example evaluated by light microscopic examination under 100× magnification with oil immersion. In addition, these micrographs show the histopathological changes at their phases of the seminiferous tubules and diverse stages in the cycle of spermatogenesis. The images were used as a handy reference for recognizing the staging and assessing the development of the testis with compare reputation of Scoring Method. Different abbreviated arrows and markings indicate the following: Z, zygotene spermatocyte; P, pachytene spermatocyte; D, diplotene spermatocytes; M, meiotic germ cell; S1–16, Step 1–16 spermatids and different markings inside the images were inserted through Adobe Photoshop CS5.

**Figure 6:**
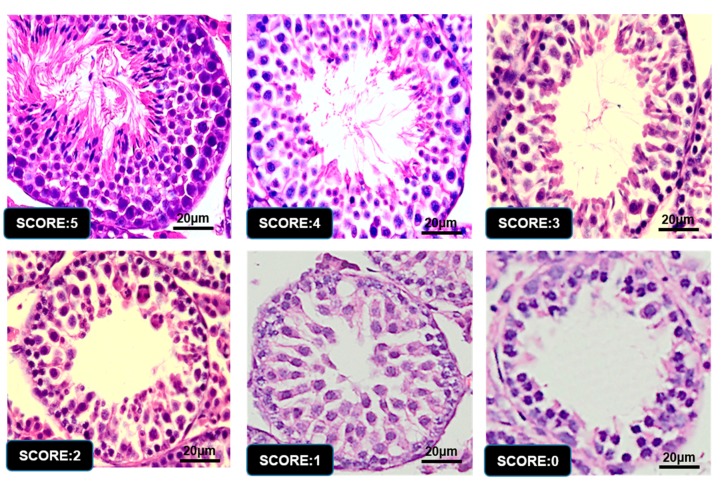
Images of seminiferous epithelia evaluation by testis scoring methodology at the age of day 35. According to the scoring, individual image obtains their respective score for having its differential characteristics of histological structure explained previously in Figure 5 & Figure 6. Images were analyzed using HE staining and were achieved by light microscope under 40× magnification.

Images in Figure 5 and Figure 6 were used as a handy reference for recognizing the stages and assessing the development of the testis considering our scoring criteria collectively (Table 1). These micrographs showed the histopathological changes at the phases of seminiferous tubules and diverse stages in the cycle of spermatogenesis (Figure 5. Hematoxylin staining of seminiferous epithelia at stages I–XII.). The arrangement micrographs of Figure 5 were evaluated by light microscopic examination under 20× magnification, presented as some common examples and helped to distinguish different type of germ cells respectively. Micrographs of Figure 6 were evaluated under 100× magnification. Special figures and characters were donated according to the staging method defined for laboratory mouse [32,37]. 

### 3.6. Graphic Explanation of Testis Scoring Method

After thoroughly observation and assessment of micrographs among different groups, the result of score for each group showed in Figure 2A. The score of “M-RJ” group mice was higher than CON group (*p* < 0.05) while a reduction of scoring in “H-RJ” group was statistically significant (*p* < 0.05) compared with CON on PNDs 21 (Figure 2A). In addition, mice supplemented with H-RJ (500 mg/kg/day) showed a significant decrease (*p* < 0.001) in testis scoring compared with CON on PND 35 (Figure 2A), which indicated that the spermatogenesis development of the testis under H-RJ treatment maybe not in healthy condition. However, the number of scoring evaluation of mice in M-RJ group was increased compared with CON group (*p* = 0.057). Corresponding to the microscopic observation and findings perspective, testis in H-RJ group seems to be less significant (*p* = 0.057).

### 3.7. Effect of Oral Administration of Royal Jelly on Proliferation in Germ Cells

**Figure 7:**
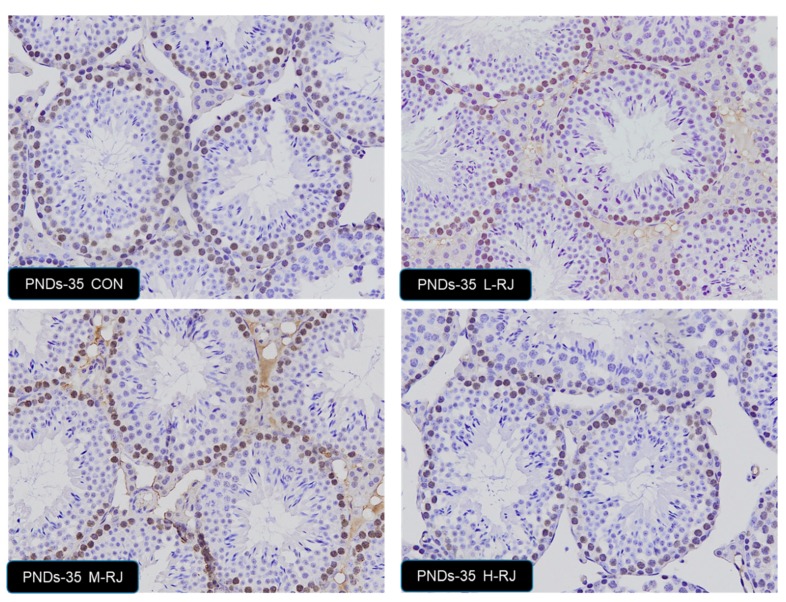
The IHC for PCNA of the testis of CON and treatment groups PNDs 35. We also detected apoptotic cells at PNDs 07, PNDs 14 and PNDs 21. However, there was no obviously significant differences among the groups in mentioned organ (data not shown). Magnification: 20×. The number of PCNA-positive germ cells were detected by IMAGE-PRO 6.0 and was analyzed by SPSS 20.0 (Version 20.0).

To explore the role of different level of dietary royal jelly on germ cell growth in testis, the proliferation detection of germ cell was carried. We used PCNA-specific antibodies to visualize the cells undergoing DNA synthesis. In CON group testis, strong PCNA positivity was detected in the spermatogonia and some types of spermatocytes, which suggest active cell proliferation (Figure 7). The PCNA-positive spermatogonia were arranged regularly and were located near the basement membrane in control group mice. Similar results were found in L-RJ and M-RJ testis. After oral H-RJ administration, less PCNA-positive cells were detected and they were not arranged regularly. The statistics result not significantly changed among the groups. The stages of PNDs 7, PNDs 14, PNDs 21 and PNDs 35 not showed statistically differences. 

### 3.8. Administration of RJ Decreases Apoptosis in the Testis

**Figure 8:**
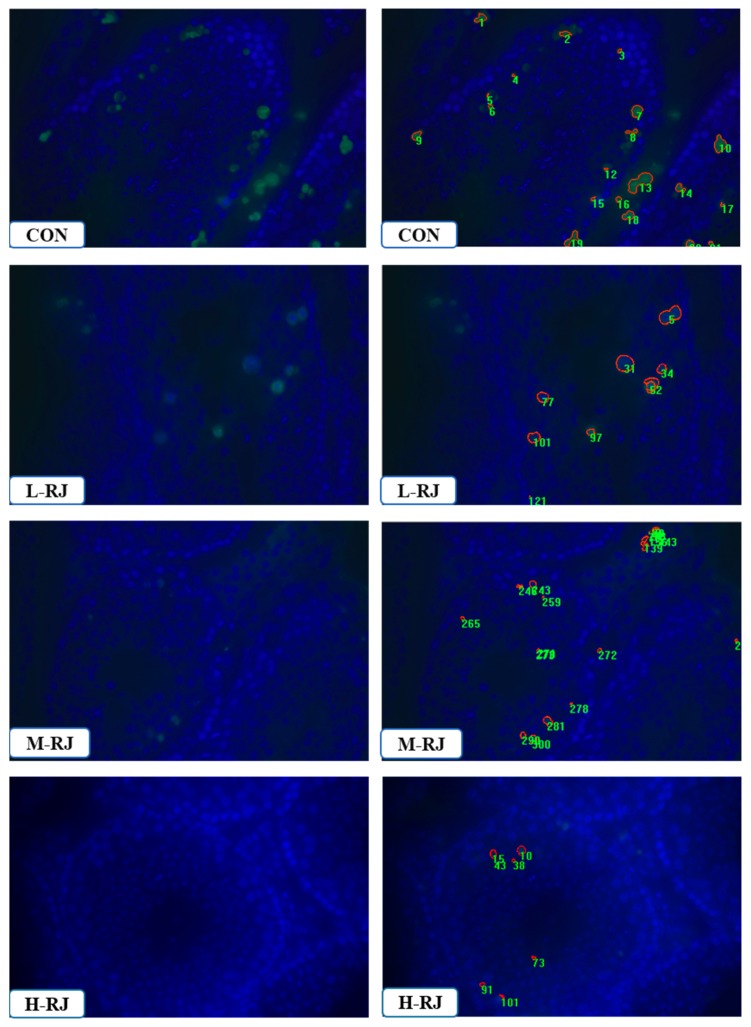
Administration of RJ reduced apoptosis in the testis. Apoptosis analysis of the CON and treatment groups. From top to bottom are the testis of CON, L-RJ, M-RJ and H–RJ, the left column showed apoptotic figures among groups, the right column were apoptotic cells in the figures which was labeled by IMAGE -PRO 6.0. Data collected using a Nikon DS-U3 system and Image-Pro Plus6.0 software. Statistical analysis was performed using Student’s t-test. Magnification: 40x.

To identify the effect of royal jelly on the testis, TUNEL-based kit was carried to detect the apoptotic cells in mouse testis. At PNDs 35, the apoptotic cells in CON mice L-RJ and M-RJ mice were located at both basement membrane and lumen of the tubules. In contrast, apoptotic cells in the testis of H-RJ mice were less detected near the basement membrane (Figure 8). Meanwhile, there were less apoptotic cells in the testis of H-RJ mice compare to the CON group (*p* < 0.05) (Figure 2C). We also detected apoptotic cells in PNDs 07, PNDs 14 and PNDs 21. However, there was no obviously significant differences among the groups in mentioned organ (data not shown). 

### 3.9. Determination of Sexual Hormones Concentration

Sexual hormones play an important role in regulation and maintenance of spermatogenesis. In current study, Follicle Stimulating Hormone (FSH), Luteinizing Hormone (LH), Estradiol (E2) and Testosterone (T) were detected at two time points (PNDs 21 and PNDs 35) by radioimmunoassay according to the manufacturer’s instructions. As shown in Figure 8, FSH level in L-RJ, M-RJ and H-RJ groups significantly decreased (*p* < 0.05) compared with the CON group at the third week (PNDs 21). Similarly, L-RJ and H-RJ treatment decreased (*p* < 0.05) the level of E2 respectively compared with the CON group at the same timing point. In addition, the level of LH and T were changed after royal jelly treatment but they were not statistically significant at two timing points.

## 4. Discussion 

Spermatogenesis and testicular development can be influenced by various effectors and can causes in deformity and low quality of sperms and poor development of testicular tissues. However, reproductive hormonal regulatory secretion is another phenomenon which can be affected apparently and very sensitive to unfavorable changes respectively. The main elucidated proteins of royal jelly are significant components of its composition, which include the royalisin, jelleines, royalactin, apisimin, apolipophorin III-like protein and glucose oxidase [3]. These proteins exhibit various biological activities. Royal Jelly has been demonstrated to possess numerous functional properties such as antibacterial activity, anti-inflammatory activity, antioxidant activity and hypotensive activities. Biological activities of RJ are mainly attributed to the bioactive fatty acids, proteins and phenolic compounds [38]. The relative biological effect of Major Royal Jelly Proteins (MRJPs) has been studied extensively in vitro, but results have much complicated for clarification, particularly in reproductive system [39,40,41]. The Major Royal Jelly Proteins (MRJPs) have multiple biological functions in addition to their role in honey bee larval development. MRJPs and royal actin are the main factors which provide the antibacterial activity to RJ especially against Gram positive bacteria [3]. 

**Figure 9:**
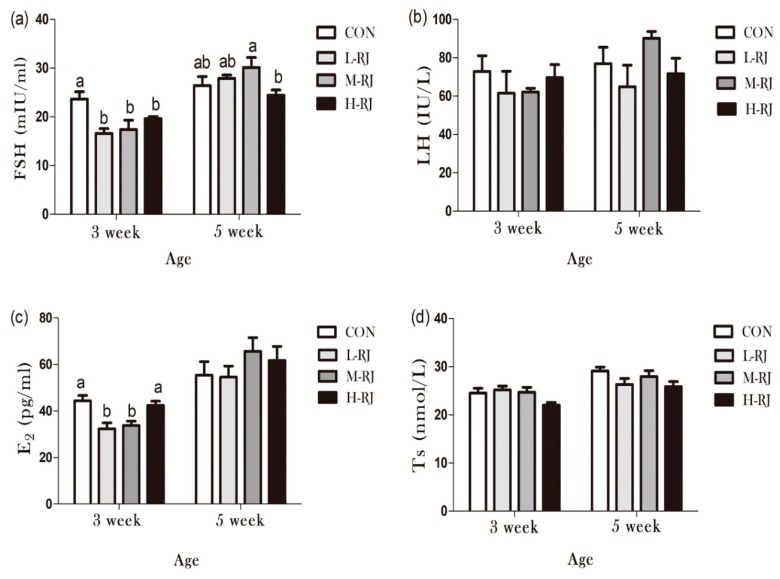
Reproductive hormones secretion level of pubescent male mice exposed to oral freeze-dried royal jelly. Data are shown as mean ± SE (n = 7) and different letters (**a**,**b**,**c**) within a column represent significant (*p* < 0.05) differences among groups. Some abbreviated letters indicate the following: Follicle Stimulating Hormone (FSH), Luteinizing Hormone (LH), Estradiol2 (E2) and Testosterone (T).

Progressive development of testis at early pubescent period was a remarkable finding in the present study. The administrated H-RJ dose have affected the value of mean tubules diameter and consequently decreased 5% than CON (Table 3). In addition, on PNDs 14, we observed that the H-RJ effected on the seminiferous tubules and enlarged approximately the lumen space than CON group (Figure 4) respectively. Moreover, the value of seminiferous epithelial height in M-RJ and H-RJ increased significantly while compared with CON on PNDs 14. It has been reported that, the formation of seminiferous tubules lumen was appeared at day 12 post-partum [42]. In our study, it was noticed that, the first wave of spermatocytes undergoing meiotic divisions was observed on PNDs 10. While, spermatocyte released into the lumen as mature sperms it become at PNDs 35. The wide of lumen can be narrow and even invisible because multiple factors regulated its development and can be affected by periods of times. For example, FSH and LH, the essential hormonal regulators of testicular functions, act through their receptors in Sertoli and Leydig cells respectively [43]. Consequently, experimental phenomena suggested that oral H-RJ may accelerate the development of some stages in reproductive system during neonatal time. Additionally, value of testis weight, mean tubule diameter and seminiferous epithelial height decreased significantly in H-RJ treatment group compared to CON at PNDs 21. Similarly, there are detectable significant decrease among the testis weight, mean tubule diameter and seminiferous epithelial height of H-RJ over CON on PNDs 35. However, these value index of M-RJ increased significantly when compared with CON on PNDs 35 (Table 2 and Table 3, Figure 2B). In comparison with two time point level of FSH in Figure 9a, treatment groups were significant decreased compared to CON at PNDs 21. However, a significant increase in L-RJ and M-RJ was observed at PNDs 35 Figure 9a. Additionally, our present study clarified that the major contribution to this abnormal results may attribute to deficient FSH secretion and immunoneutralization, which may lead to decreased in germ cell apoptosis [44,45]. Moreover, our experimental results are consistent and confirm the apoptosis in testicular cells (Figure 2C). The antioxidant properties of RJ may be a potential reason for the reduction of apoptosis in germ cells. 

This study also revealed that low and moderate doses of freeze-dried RJ enhanced testicular development and spermatogenesis in pubescent mice. The effect could be also attributed to various valuable nutritive substances, such as vitamin C, vitamin E and arginine as RJ contents [46]. 

Apparently, enhancements in reproductive functions following RJ treatments can also be attributed to different amino acids and 10- hydroxy -2- decanoic acid/or some essential vitamins [47]. Taking this to consideration, it has also been reported that feeding rabbits with different doses of vitamin C, reduced abnormal sperms and improved sperms concentration owing its antioxidant properties [48,49]. The present study showed that freeze-dried RJ, abnormalized the testicular structure and disordered the sexual hormones secretion level in pubescent mice. We measured the mice body weight on weekly bases, although different doses of treatment showed no significant changes on growth performance, particularly moderate dose enhanced the testicular development. Interestingly, significant changes happened to the value of testis weight, the mean tubules diameter as well as seminiferous epithelial height respectively. 

Due to imperative role of testosterone in spermatogenesis, previous study has demonstrated that feeding golden hamster with RJ results in higher testosterone synthesis than control group in a dose dependent manner and indicated that long-term RJ feeding inhibits testicular dysfunction [43]. The RJ also contains l-arginine and carnitine amino acids which are essential for spermatogenesis [50].

Apparently, FSH increases cAMP levels of inhibin and it keeps an inversely correlated relationship with serum FSH [51].

Our data illustrated that freeze-dried RJ elicits estrogen-like effects and it affects the development of reproductive system (Table 2 and Figure 7). We combined all the data at different cross time points (Table 2 and Table 3), neither the weight nor the mean tubules diameter had significant changes on PNDs 7, there was no detectable significant difference among treatment groups and CON in seminiferous epithelial height on PNDs 7 (Figure 2B),but the data indicated that both may be important because pups are sensitive to the estrogen-like compounds, which have animal and plant sources [27]. Our study confirms that freeze-dried RJ is rapidly hydrolyzed and absorbed in to neonatal blood circulation because 10-HDA only found in RJ and as a quality marker of RJ is proved to have the same effect compared with only RJ administration [25,32,41]. Therefore, metabolic deficiency maybe the potential reason.

The hypothalamic pituitary–gonadal (HPG) axis controlled by the central nervous system is the humoral component of an intercommunicating neural and endocrine system. Regulation of fertility is one of its most important functions [52]. Oral exposure to RJ, which has known with estrogenic activity in prepubertal and mature rodents and in the adult human [53,54]. 

In the last recent years, the role of estrogen in the males has been re-considered broadly. The physiological virtuous function of this sexual hormone, typically female hormone regulates the function of epididymis and development of the spermatogenesis in males respectively [55,56]. Many Endocrine Disruptor Chemicals (EDC) with estrogen-like action interfere with the male reproductive physiology causing skewed sex ratio, diminished sperm count, reduced sexual behavior, abridged spawning and fertilization in many life species [57,58]. Our results clearly demonstrated the effects of freeze-dried royal jelly on estradiol concentration, estrogen level was significantly reduced with low and moderate doses in PNDs 21 and increased by moderate and high doses in PNDs 35 of freeze-dried royal jelly oral exposure among the groups. Apparently, in some organism’s estrogen showed severe unfavorable effects such as disruption of reproductive cycle, testicular growth inhibition, abnormal morphology and changes in sex ration and sex reversal effects. Furthermore, it has been reported that, RJ contain testosterone hormone and have steroid hormone-type activities respectively [59,60,61]. LH and FSH are essential regulators of testicular function, LH stimulates production of testosterone by the Leydig cells, which exerts sexual and anabolic action in return. There are evidences that testosterone is crucial for male genital differentiation and growth, and it can complete spermatogenesis relatively independent of FSH [62], which was consistent with the previous studied carried out on recombinant FSH on Ts induced spermatogenesis gonadotrophin-deficient mice [63,64]. Although administration of FSH together with LH or Testosterone (T) augments the supportive role of LH or (T) on spermatogenesis. However, FSH individually effect the formation of elongated post-step 8 spermatids [65,66]. These studies support the idea that limited Testosterone (T) secretion and FSH deficiency Figure 9a,d) may have adverse consequences such as reduced testicular size e.g., (weight) (Table 2, PNDs 21, PNDs 35) and irrational histoarchitecture of seminiferous tubules Figure 3. Collectively, our results demonstrate that, according to the Johnsen score criterion, testis in M-RJ group exceedingly developed significantly (*p* < 0.05) compare to CON group after histologically evaluation, L-RJ group held small margin of difference with CON group, while H-RJ group was more underdeveloped at the dosage of 500 mg/kg/day significantly (*p* < 0.01). Our finding suggests that, these effects were similar to previous studies at similar doses [67]. In summary, our study demonstrates that exposure to oral freeze-dried RJ during neonatal development generates adverse consequences in the adult male mouse reproductive system respectively. However, dual character performance of freeze-dried RJ suggests that risk to child reproductive system health should be carefully consider, especially freeze-dried RJ is an easily accessible honey products with good reputation in daily life. Hence, the present study model is a suitable dose of freeze-dried RJ and further researches are required to investigate in neonatal mice.

## 5. Conclusions

In summary, exposure to freeze-dried Royal Jelly for 35 consecutive days enhanced testis growth performance and spermatogenesis with low and moderate doses while high dose impaired its development. The current study demonstrates that, oral administration of freeze-dried royal jelly had adverse effects on some sexual hormone regulatory secretions in reproductive tract and cause adverse effects in hormones secretions in return. Additionally, high dose of RJ can reduce apoptosis of germ cells significantly via acceleration of reduced testis size and deteriorates the spermatogenesis. Our data provide a novel perspective in understanding the interaction of royal jelly via oral administration on testicular development and hormone regulations. These insights might pave the way for deeper investigations for studying physiological mechanism of royal jelly in mice reproductive tract and would have a great impact on reproduction consequences.

## Figures and Tables

**Table 1:** Criteria for scoring the spermatogenesis.

5	Complete spermatogenesis and perfect tubules
4	Spermatozoa present with disorganized spermatogenesis
3	No spermatozoa, but long spermatids present
2	No long spermatids present, but round spermatids present
1	No spermatids, but spermatocytes present
0	Only spermatogonia present

**Table 2:** Organs weight and pH measurement at different Post-Natal Days.

Group	Day	Testis (mg)	Epididymis (mg)	Spleen (mg)	Liver (mg)	Kidney (mg)	pH
	PNDs-07	16.66 ± 1.50	8.21 ± 0.02	34.61 ± 1.56 ^a^	200 ± 0.02	73.94 ± 3.96	3.49 ± 0.21
Control	PNDs-14	33.48 ± 1.26 ^b^	13.12 ± 0.33	39.27 ± 3.83 ^a^	250 ± 0.02 ^b^	104.10 ± 2.64 ^b^	4.31 ± 0.07
	PNDs-21	77.16 ± 2.72 ^bc^	16.96 ± 0.64	120 ± 0.01 ^b^	850 ± 0.03 ^b^	205.64 ± 7.88 ^b^	5.05 ± 0.26 ^a^
	PNDs-35	166.54 ± 7.22 ^b^	38.94 ± 1.60	100 ± 0.01 ^b^	1480 ± 0.11	380 ± 0.02	3.59 ± 0.05 ^c^
	PNDs-07	15.02 ± 0.92	11.21 ± 0.01	30.10 ± 1.23 ^b^	210 ± 0.01	78.58 ± 2.00	3.67 ± 0.24
Low dose	PNDs-14	35.00 ± 1.78 ^b^	14.12 ± 0.31	41.16 ± 3.22 ^a^	260 ± 0.01 ^b^	108.60 ± 5.64 ^ab^	4.38 ± 0.06
	PNDs-21	80.84 ± 2.62 ^ab^	17.68 ± 1.10	130 ± 0.02 ^b^	840 ± 0.02 ^b^	234.28 ± 4.38 ^a^	4.81 ± 0.07 ^b^
	PNDs-35	163.70 ± 5.92 ^b^	40.20 ± 0.80	100 ± 0.02 ^b^	1510 ± 0.03	340 ± 0.027	4.91 ± 0.12 ^a^
Moderate Dose	PNDs-07	17.8 ± 0.62 ^b^	13.21 ± 0.25	22.97 ± 1.00 ^c^	150 ± 0.01	73.46 ± 3.02	3.84 ± 0.21
	PNDs-14	40.72 ± 2.24 ^a^	16.12 ± 0.43	48.76 ± 4.35 ^a^	320 ± 0.01 ^a^	118.60 ± 0.94 ^a^	4.37 ± 0.09
	PNDs-21	81.20 ± 1.58 ^a^	19.16 ± 0.94	100 ± 0.01 ^b^	730 ± 0.02 ^c^	175.20 ± 3.18 ^c^	4.09 ± 0.43 ^b^
	PNDs-35	173.34 ± 7.72 ^a^	47.20 ± 1.50	120 ± 0.02 ^ab^	1760 ± 0.09	400 ± 0.10	4.42 ± 0.05 ^b^
	PNDs-07	16.52 ± 1.02	9.21 ± 0.26	32.36 ± 1.42 ^ab^	180 ± 0.01	70.06 ± 3.38	3.79 ± 0.22
High dose	PNDs-14	34.26 ± 1.30^b^	15.12 ± 0.48	28.28 ± 2.65 ^b^	270 ± 0.01 ^b^	97.76 ± 6.62 ^b^	4.21 ± 0.06
	PNDs-21	79.36 ± 3.46 ^b^	18.60 ± 1.28	160 ± 0.01 ^a^	920 ± 0.01 ^a^	206.2 ± 6.22 ^b^	5.09 ± 0.16 ^a^
	PNDs-35	125.34 ± 10.62 ^c^	41.80 ± 5.56	120 ± 0.01 ^a^	1650 ± 0.10	360 ± 0.04	4.40 ± 0.03 ^b^

Testis, Epididymis, spleen, liver and kidneys weight unit (mg, n = 7); the table shows different treatments, low dose 125 mg/kg/day body weight/day “Low dose-RJ”, moderate dose 250 mg/kg/day body weight/day “Moderate-RJ”, High dose 500 mg/kg/day body weight/day “High dose-RJ” and Control “Control”. pH (potential of Hydrogen) determine the acidity level of the stomach in different time points. Values shown are mean ± SE. (Standard Errors), Different letters a, b, c within a column denote significant differences between treatments significantly (*p* < 0.05).

**Table 3:** Effect of different dosages of freeze-dried RJ on mean seminiferous tubules diameter of male mice.

	CON	L-RJ	M-RJ	H-RJ	*p*-Value
**PND 07**	64.04 ± 0.68	63.52 ± 0.79	65.84 ± 1.01	63.86 ± 1.30	0.36
**PND 14**	74.56 ± 1.31b	74.84 ± 2.06b	87.34 ± 1.08a	70.90 ± 0.68c	0.0003
**PND 21**	153.17 ± 3.54a	150.64 ± 2.72a	152.27 ± 2.25a	134.95 ± 1.89b	0.002
**PND 35**	189.99 ± 3.63ab	185.67 ± 2.24b	193.89 ± 3.37a	173.10 ± 2.87c	0.02

Values are expressed as mean ± SEM (n = 7). Different letters (a, b, c) within a column denote significant (*p* < 0.05) differences between treatments. “CON” means the control group, “L-RJ”, “M-RJ” and “H-RJ” are the abbreviation of “low dosage of RJ”, “moderate dosage of RJ” and “high dosage of RJ”, and “PNDs” is abbreviated for Post Natal Days.
